# Delineating the role of FANCA in glucose-stimulated insulin secretion in β cells through its protein interactome

**DOI:** 10.1371/journal.pone.0220568

**Published:** 2019-08-28

**Authors:** Dragana Lagundžin, Wen-Feng Hu, Henry C. H. Law, Kimiko L. Krieger, Fangfang Qiao, Emalie J. Clement, Andjela T. Drincic, Olgica Nedić, Michael J. Naldrett, Sophie Alvarez, Nicholas T. Woods

**Affiliations:** 1 Eppley Institute for Research in Cancer and Allied Diseases, Fred & Pamela Buffett Cancer Center, University of Nebraska Medical Center, Omaha, Nebraska, United States of America; 2 Mass Spectrometry and Proteomics Core Facility, University of Nebraska Medical Center, Omaha, Nebraska, United States of America; 3 Department of Internal Medicine: Diabetes, Endocrinology and Metabolism, University of Nebraska Medical Center, Omaha, Nebraska, United States of America; 4 Institute for the Application of Nuclear Energy, University of Belgrade, Banatska, Belgrade, Serbia; 5 Proteomics & Metabolomics Facility, Nebraska Center for Biotechnology, University of Nebraska–Lincoln, Nebraska, United States of America; Universität Regensburg, GERMANY

## Abstract

Hyperinsulinemia affects 72% of Fanconi anemia (FA) patients and an additional 25% experience lowered glucose tolerance or frank diabetes. The underlying molecular mechanisms contributing to the dysfunction of FA pancreas β cells is unknown. Therefore, we sought to evaluate the functional role of FANCA, the most commonly mutated gene in FA, in glucose-stimulated insulin secretion (GSIS). This study reveals that FANCA or FANCB knockdown impairs GSIS in human pancreas β cell line EndoC-βH3. To identify potential pathways by which FANCA might regulate GSIS, we employed a proteomics approach to identify FANCA protein interactions in EndoC-βH3 differentially regulated in response to elevated glucose levels. Glucose-dependent changes in the FANCA interaction network were observed, including increased association with other FA family proteins, suggesting an activation of the DNA damage response in response to elevated glucose levels. Reactive oxygen species increase in response to glucose stimulation and are necessary for GSIS in EndoC-βH3 cells. Glucose-induced activation of the DNA damage response was also observed as an increase in the DNA damage foci marker γ-H2AX and dependent upon the presence of reactive oxygen species. These results illuminate the role of FANCA in GSIS and its protein interactions regulated by glucose stimulation that may explain the prevalence of β cell-specific endocrinopathies in FA patients.

## Introduction

Fanconi anemia is a rare disease with 22 complementation groups representing mutations in individual genes. Numerous abnormal physical and molecular phenotypes are associated with this disease, most notably bone marrow failure (BMF), acute myelogenous leukemia (AML) and a spectrum of other malignancies that contribute to patient mortality. Approximately 90% of FA patients will experience BMF as their first hematopoietic presentation of disease and an AML incidence rate of 33% by age 40 [1]. FA patients also display a spectrum of congenital defects, such as microcephaly, malformed or absent thumbs, short stature, and skin discolorations [2]. Up to one-third of FA patients exhibit no physically discernable characteristic. Advances in hematopoietic cell transplant (HCT) therapy in FA patients have significantly reduced the mortality associated with AML [2, 3], yet these patients remain prone to a spectrum of cancers including breast, head and neck, and genitourinary cancers [4].

In addition, 80% of all FA individuals exhibit at least one endocrine abnormality, such as growth hormone deficiency, abnormal glucose or insulin metabolism, dyslipidemia, hypothyroidism, hypogonadism, or infertility [5]. The prevalence of diabetes in FA patients is 8–10%, and up to 68% of FA patients exhibit impaired glucose tolerance [5–10]. Both FA and the treatment of its associated BMF with hematopoietic cell transplantation (HCT) increase the risk of developing diabetes [11–13]. It was also found that 25% of post-HCT FA patients have reduced first-phase insulin release [14], which may lead to diabetes development. However, FA patients have a high likelihood of developing diabetes even before HCT [9, 10], suggesting the underlying cause of the elevated rates of endocrinopathies in FA patients is not entirely related to these treatments. Islet cell death can be caused by chronic hyperglycemia, referred to as glucose toxicity [15–17]. FA patients have a propensity for hyperglycemia that originates due to either β cell dysfunction or insulin resistance [5]. The cause of these abnormalities is unknown but has been postulated to be caused by several factors, including increased reactive oxygen species (ROS)-mediated damage to β cells [18–20], iron overload in patients receiving transfusions, or medications commonly used to treat FA patients (androgens & corticosteroids) [5]. Therefore, it is reasonable to assume that FA proteins possess undiscovered functions in β cells that might affect insulin secretion. Understanding the role of FA proteins in pancreas β cell function would help develop strategies to reduce this prevalent co-morbidity in FA patients.

Controlling pancreatic endocrinopathies is important for the overall health of FA patients because it has the potential to prevent adverse outcomes to therapies used to treat FA [9]. For instance, hyperglycemia negatively impacts clinical outcome and survival after HCT through increased risk of septicemia, graft versus host disease, and death in pediatric and adult patients [21–24]. Current therapies for FA patients with normal fasting glucose but impaired glucose tolerance show varying levels of success [5]. Therefore, significant research efforts are needed to understand the molecular mechanisms by which FA mutations predispose these patients to endocrinopathies so that improved treatment and monitoring protocols can be developed to reduce these comorbidities and enhance FA patient outcomes after HCT.

The aim of this study was to evaluate the role of FANCA in GSIS and evaluate its interactome in pancreas β cells and the effect of glucose stimulation on these interactions. GSIS is controlled by many factors including non-glucose nutrients, hormones, and neural inputs, but can be measured by analyzing the amount of insulin secreted from β cell following glucose stimulation [25]. In this study, we have determined that inhibition of FANCA or FANCB prevents GSIS in EndoC-βH3 cells. To evaluate the role of FANCA in GSIS, we established the protein-protein interactome of endogenous FANCA in both EndoC-βH3 cells and identify β cell-specific FANCA interactions differentially regulated by glucose stimulation, including the assembly of the FA complex. Furthermore, we demonstrate that ROS production is required for insulin secretion in EndoC-βH3 cells and responsible for the increase in DNA damage marked by γ-H2AX levels and the assembly of FA protein interactions with FANCA following glucose stimulation.

## Materials and methods

### Cell lines

Human embryonal kidney 293FT cell line (ThermoFisher) was cultured in Dulbecco’s Modified Eagle Medium (DMEM) (Corning), containing 10% fetal bovine serum (Atlanta Biologicals) and 1% penicillin-streptomycin (Corning). Human pancreatic islet EndoC-βH3 cell line was obtained from Univercell-Biosolutions. Cells were seeded at the density of 70000/cm^2^ onto Matrigel (4 μl/cm^2^)-fibronectin (2 μg/cm^2^)-coated TPP plates (Midwest Scientific) and cultured in Optiβ1 medium (Univercell-Biosolutions), containing 10 μg/mL puromycin and 1% penicillin-streptomycin. Cells were treated with 1 μM 4-hydroxytamoxifen, twice a week for the period of 3 weeks, per the supplier’s instructions. After 3 weeks of treatment, complete cell proliferation arrest was observed, and tamoxifen was removed from the medium and all following experiments were conducted within the following 2 weeks. Human pancreatic cancer MiaPaCa-2 (CRL-1420) cell line (ATCC) was cultured in DMEM, containing 10% fetal bovine serum, 2.5% horse serum and 1% penicillin-streptomycin.

### Co-immunoprecipitation of FANCA complexes

Cells were lysed using NETN lysis buffer with the protease and phosphatase inhibitors as described above. The volume of buffer used for each sample was 3 times of the cell pellet size. Sample protein concentrations were measured using BCA Protein Assay kit (ThermoFisher). Identification and analysis of FANCA interacting proteins in 293FT, EndoC-βH3 and MiaPaCa-2 cells was done using Dynabeads Antibody Coupling Kit (ThermoFisher). Anti-FANCA antibodies (60 μg) (Bethyl Laboratories) were coupled onto magnetic beads (3 mg) at 37 ºC overnight, with rotation. The same amount of rabbit polyclonal IgG (Santa Cruz Biotechnology) was coupled to beads and used as a negative control. Beads were thoroughly washed with buffers provided within the kit and incubated with 3 mg of proteins from the cell lysates for 2 h at 4°C, with rotation. To remove the non-specific binders, beads were washed 4 times with NETN buffer containing 50 mM NaCl and 1 mM MgCl_2_. Then, they were washed once with NETN containing 0.02% Tween, for 5 min with rotation. To elute the bound protein fraction, beads were incubated with 500 μL 0.5 M NH_4_OH/0.5 mM EDTA, pH 11.0, for 10 min with rotation. The eluted fraction was neutralized with acetic acid, concentrated using a SpeedVac and submitted to gel electrophoresis.

### Protein electrophoresis

For the mass spectrometry experiments, 10% Criterion XT Bis-Tris Protein Gels (Bio-Rad) were used for the protein electrophoretic separation with a compatible XT MOPS (3-(N-morpholino)propanesulfonic acid) running buffer (Bio-Rad). For the immunoblot experiments, sodium dodecyl sulfate polyacrylamide gel electrophoresis (SDS-PAGE) using either 6% or 12% gels was used for the protein separation and detection.

### Protein in-gel digestion

After electrophoretic protein separation, gels were stained with Coomassie Brilliant Blue G-250 dye (ThermoFisher) for 2 h and left to destain overnight in the destain solution (10% acetic acid/20% methanol). The destained gel pieces were incised, washed with HPLC water and dehydrated with neat acetonitrile (ACN). Proteins were reduced with 2 mM Tris(2-carboxyethyl)phosphine (TCEP) in 50 mM ammonium bicarbonate (NH_4_HCO_3_, AmBic) for 1 h at 37°C and then dehydrated with ACN. The reduced proteins were alkylated with 50 mM iodoacetamide (IAA)/50 mM AmBic, for 20 min in dark with rotation. The gel pieces were dehydrated again with ACN to remove all the reagents. MS-grade trypsin (10 ng/μL) (Promega) was added to the samples and incubated for 30 min on ice. After the excess of trypsin was removed from tubes, 25 mM AmBic was added to cover the gel pieces and incubate overnight at 37°C. Digested peptides were then extracted from the gel with 50% ACN/0.1% trifluoroacetic acid solution. Samples were dried in a SpeedVac, re-dissolved in 15 μL 0.1% formic acid and submitted for LC-MS/MS analysis.

### Mass spectrometry

In-gel digested peptide samples were analyzed using either the Orbitrap Elite, Orbitrap Q Exactive HF, or Orbitrap Fusion Lumos. The Q Exactive HF and Fusion Lumos instruments were coupled with UltiMate 3000 RSLCnano LC systems (Thermo Scientific) and the Elite with the Eksigent Ultra NanoLC 2D with nanoflex cHiPLC. For the Q Exactive HF, online peptide separation was carried out by first desalting peptides for 3 min on a trapping column (C18 Pepmap100 0.3x5 mm, 5 μm, 100A) at 5 μL/min in 1% acetonitrile, 0.1% formic acid before separation into the mass spectrometer using a 75 μm x 25 cm peptide CSH C18 130A, 1.7 μm nano-column (Waters Corp, Milford, MA) using a linear gradient run at 300 nL/min from 4% B to 45% B over 85 min. Solvents: A is water + 0.1% formic acid, and B is 80% ACN + 0.1% formic acid. The total run time was 105 min. The Q Exactive HF was run in a top 12 data-dependent acquisition mode triggering on peptides with charge states 2 to 4 over the mass range of 197–1500 *m/z* with MS1 resolution set to 120,000, AGC target of 3e^6^ and a maximum ion time of 60 ms. MS2 resolution was set to 15,000, AGC target of 1e^5^ and a maximum ion time of 250 ms. Dynamic exclusion was 12 s for a low complexity sample, quadrupole isolation width 1.6 *m/z*, spray voltage 1.9 kV, capillary temperature 275°C and a normalized collision energy of 28. The minimum AGC target and the intensity threshold were set to 5e^3^ and 2e^4^, respectively. For analysis with the Orbitrap Elite, around 5 μL (500 ng) of each sample was loaded on a cHiPLC column (0.5 mm C18 CL 3 μm 120 Å trap column, Eksigent) using 0.1% formic acid and fractionated with an analytical column (75 μm x 15 cm C18 CK 3 μm 120 Å ChromXP, Eksigent). The samples were eluted using a 60 min linear gradient of ACN (0–60%) in 0.1% formic acid. The parameters for the method created were: nanospray needle voltage in positive mode: 2100 V; LC flow rate: 300 nL/min; Orbitrap scan mode was used for MS/MS, with the resolution 120.000 and the scan range 300–2000 *m/z*. Peptides were put into dynamic exclusion for 15 s after detected 2 times. Fragmentation method: CID; Precursor ion isolation width: 1.0 *m/z*; Maximum injection time for MS/MS: 100 ms. For analysis with the Lumos, around 5 μL (500 ng) of each sample was loaded onto the trap column (Acclaim PepMap 100, 75 μm × 2 cm, nanoViper, Thermo Scientific) using formic acid (0.1%) and resolved in the rapid separation liquid chromatography (RSLC) column (Acclaim PepMap RSLC C18, 75 μm × 15 cm, nanoViper, Thermo Scientific). The samples were eluted using an 85 min linear gradient of ACN (4–45%) in 0.1% formic acid. The parameters for the method created for all Lumos experiments were as follows: nanospray needle voltage in positive mode: 1950 V; column flow rate: 300 nL/min and loading pump flow: 1.5 μL/min for 20 min; Inject mode: μL PickUp. Orbitrap scan mode was used for MS, with the resolution 120.000 and the scan range 375–1500 *m/z*. Peptides were put into dynamic exclusion for 30 s after detected twice. Detector type for MS/MS was set to: Orbitrap, with the resolution: 30000, isolation mode: quadrupole (isolation window of 1.6 Da), activation type: HCD, HCD collision energy: 40%, first mass: 110 *m/z*. Stainless steel emitters were purchased from ThermoFisher (O.D. 150 μm, I.D. 30 μm, 40 mm length, inserted in a 1/32 microsleeve for installation).

### Database searching and interactome analysis

The MS/MS spectra from the peptides were searched against the Swiss-Prot database (*Homo sapiens*, downloaded at 07/2018, no. of entries = 20387) using MASCOT search engine (Matrix Science Inc, v2.5.1) [26]. Parameters on MASCOT were set as follows: Enzyme: trypsin, Max missed cleavage: 2, Peptide charge: 1+ and above, Peptide tolerance: ± 0.1 Da, Fixed modifications: carbamidomethyl (C), Variable modifications: oxidation (M), phospho (ST) and phospho (Y), MS/MS tolerance: ± 0.5 Da, Instrument: ESI-FTICR. MASCOT results for different gel cuts of the same sample were combined and analyzed using Scaffold (Proteome Software Inc., v4.5.1), which allows multiple search results to be consolidated into a single result file. 99% protein and 50% peptide thresholds were set for 293FT analysis and 99% protein and 95% peptide thresholds for EndoC-βH3 cells. These settings gave 0.1 and 0.4% protein FDR for 293FT and EndoC-βH3 datasets, respectively, and 0.0% peptide FDR for both datasets. The interaction specificity between the bait and prey proteins was assessed with the Significance Analysis of INTeractome (SAINT) algorithm [27], as previously described [28]. All experimental replicates were included in the proteomic analysis. The molecular interaction networks and functional enrichment analysis were performed using ClueGO plug-in in Cytoscape (3.6.1). ClueGO was used to analyze functional enrichments using the Reactome Pathways (20.11.2017) and Reactome Pathways (20.11.2017) annotations (Min #Gene = 3, Min %Genes = 15%, Kappa Score = 0.4, Two-sided hypergeometric test, Benjamini-Hochberg p-value correction). ProHits-viz was used to visualize the output from SAINT [29].

### shRNA-mediated silencing of Fanconi anemia proteins

The shRNA sequences from The RNA Consortium library (Sigma-Aldrich) that were used for the knockdown experiments by lentiviral transduction were either non-targeting scrambled control shScr (SHC016), FANCA targeting (#1—TRCN0000296799; #2 –TRCN0000291182), or FANCB targeting (#1 –TRCN0000160916; #2 –TRCN0000163896).

### Insulin release assay and glucose/NAC treatment

EndoC-βH3 cells were seeded onto Matrigel-fibronectin coated 12-well TPP plates (MidSci) at the density of 10^5^ cells/cm^2^. Four days after cell seeding, lentiviral-mediated knockdown of FANCA and scrambled control was conducted using the corresponding shRNAs. The next day, cells were incubated overnight with the glucose-starving Optiβ2 medium (Univercell-Biosolutions). Medium was replaced after 24 h with Krebs-Ringer Buffer (KRB-B) (116 mM NaCl, 5.06 mM KCl, 1.01 mM CaCl_2,_ 1.01 mM MgCl_2_, 1.19 mM KH_2_PO_4_, 23.96 mM NaHCO_3_, 0.1% BSA, pH 7.4) and incubated for 1 h. Cells were next incubated either with KRB-B3 (KRB-B with 5 mM glucose), KRB-B3N (KRB-B3 with 5 mM NAC), KRB-B20 (KRB-B with 20 mM glucose) or KRB-B20N (KRB-B20 with 5 mM NAC) buffer, for 1 h. The medium from each well was transferred to a micro tube and centrifuged at 700 x g for 5 min at 4°C. Insulin secretion was measured in the supernatant using STELLUX Chemi Human Insulin ELISA kit (Alpco) per manufacturer’s instructions. For the intracellular insulin content measurement, cells were lysed with NETN lysis buffer (250 mM NaCl, 5 mM EDTA, 0.5% Nonidet P-40, 50 mM Tris-HCl, pH 8.0) containing protease inhibitor cocktail (Sigma-Aldrich) and phosphatase inhibitors (50 mM NaF, 10 mM β-glycerophosphate, 0.1 mM NaVO_4_), prior to ELISA assay. Differences between the insulin concentrations were assessed by either Student’s t-test or ANOVA using the GraphPad Prism v7.03.

### Cellular ROS detection assay

DCFDA Cellular ROS detection assay kit (Abcam) was used to measure the production of ROS in the β islet cells at both 5 mM and 20 mM glucose conditions. EndoC-βH3 cells were seeded onto 96-well microplate at the density of 4 × 10^5^/well and allowed to attach overnight. Cells were incubated with glucose-starving Optiβ2 medium for 24 h and then incubated with either KRB-B3 or KRB-B20 buffer (see section Insulin Release Assay and Glucose/NAC treatment) for 1 h. 150 μM tert-butyl hydrogen peroxide (TBHP) solution provided with the kit was used as a positive control. Cells were next stained with 20 μM DCFDA for 45 min at 37°C and the fluorescence signal was read on a microplate reader at Ex/Em: 485/535.

### Immunoblot and antibodies

Primary antibodies used were FANCA (A301-980A) (1:2000), FANCD1 (A300-005A) (1:1000), FANCD2 (A302-174A) (1:2000), FANCE (A302-125A) (1:1000), FANCI (A300-212A) (1:700), FANCM (A302-637A) (1:1000), FANCO (A302-645A) (1:1000), FANCP (A302-270A) (1:1000), FANCQ (A301-315A) (1:1000), H2AX (A300-082A) (1:5000) from Bethyl Laboratories; FANCF (PA5-18202) (1:1000), FANCG (PA5-27117) (1:1000), FANCL (PA5-19332) (1:1000), PIK3C2A (MA526505) (1:1000), and TFRC (136800) (1:1000) were from ThermoFisher; β-actin (sc-47778) (1:2000) was from Santa Cruz Biotechnology; FANCS (OP92-100UG) was from Calbiochem; γ-H2AX (NB100-78356) (1:5000) was from Novus Biologicals. Secondary antibodies used were goat anti-rabbit IgG-HRP (sc-2357) (1:5000), goat anti-mouse IgG-HRP (sc-2005) (1:5000), and goat anti-rat IgG-HRP (sc-2303) (1:5000) were from Santa Cruz Biotechnology.

## Results

### GSIS is impaired following the knockdown of FANCA in human pancreatic EndoC-βH3 cells

**Fig 1:**
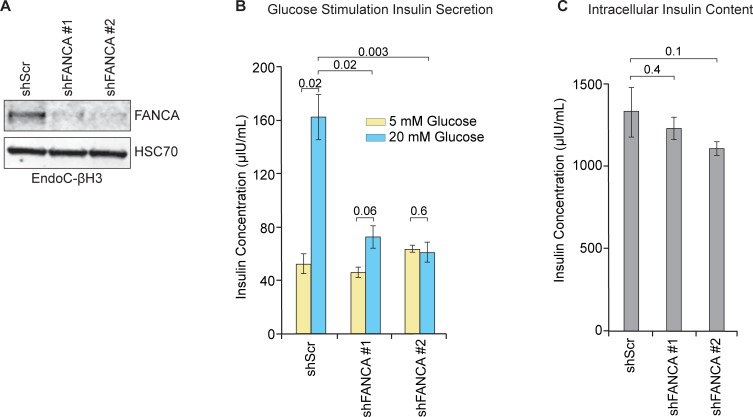
Inhibition of FANCA expression disrupts β cell GSIS. **A.** Confirmation of FANCA knockdown by shRNA (shFANCA #1 and #2) compared to non-targeting scrambled control shRNA (shScr) in EndoC-βH3 cells. **B.** GSIS profiles in EndoC-βH3 shScr or shFANCA transduced cells. Paired two-tailed Student’s t-test *p*-values are displayed in the indicated comparisons above the graph. n = 3, mean +/- standard error of the mean (SEM). **C.** Intracellular insulin content of shScr or shFANCA EndoC-βH3 cells used in the GSIS assays in panel B. Paired two-tailed Student’s t-test *p*-values are displayed in the indicated comparisons above the graph. n = 3, mean +/- SEM.

For the purposes of this study, we chose to focus on the FANCA protein, because this gene is mutated in the majority of FA cases [31]. The EndoC-βH3 pancreas beta islet cell line was generated by introducing a tamoxifen-inducible CRE-ERT2 construct into the conditionally immortalized EndoC-βH2 cell line. This led to the development of a β cell line that can be expanded in culture then reverted to a glucose-responsive, insulin-secreting, proliferation-arrested β cell with tamoxifen treatment [32]. The EndoC-βH3 is a clear improvement over other human β cell lines, such as the 1.1B4 cell line generated by electrofusion with pancreatic ductal adenocarcinoma cell line PANC-1 [33]. To investigate whether FANCA in EndoC-βH3 cells affects GSIS, two FANCA shRNAs were identified capable of knocking down this protein. Lentivirus shRNAs were generated in 293FT cells and used to transduce EndoC-βH3 cells. The shRNAs targeting FANCA (shFANCA #1 and #2) were confirmed by western blot of the EndoC-βH3 cell lysates compared to the non-targeting scrambled control (shScr) (**Fig 1A**). The mean normal blood glucose levels in humans is approximately 5.5 mM. Therefore, cells were cultured in either normal glucose (5 mM) or glucose-stimulated (20 mM) concentrations for a 1 h treatment period. The results showed that GSIS is significantly impaired after knockdown of FANCA (**Fig 1B**). The baseline insulin secretion at 5 mM glucose conditions in the shFANCA cells did not significantly differ from that of control shScr cells. We observed no differences in the intracellular insulin content after the knockdown of FANCA (**Fig 1C**). Together, these results suggest the defect in GSIS caused by depletion of FANCA may be attributed to the disruption of pathways regulating insulin secretion.

### Optimization of the purification and analysis process to evaluate endogenous FANCA protein-protein interactions in 293FT cells

**Fig 2:**
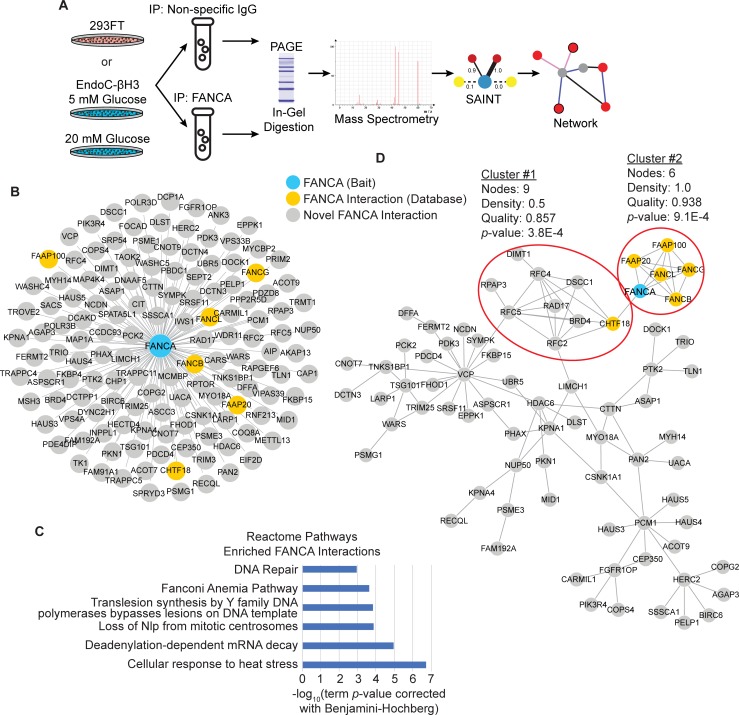
Optimization and analysis of the FANCA PPIN in 293FT cells. **A.** Overview of experimental design to determine the FANCA PPIN in 293FT and EndoC-βH3 cells. All experiments were performed as biological duplicates. **B.** 137 FANCA protein-protein interactions identified from 293FT lysates. Blue node = FANCA bait, Grey nodes = prey proteins interacting with FANCA with a SAINT-determined BFDR ≤ 0.05 and SAINT score ≥ 0.8. **C.** ClueGO analysis of enriched Reactome Pathways using the 137 high confidence FANCA interactions identified in 293FT. **D.** Known protein interactions from database imputed on the set of 137 proteins interacting with FANCA with BisoGenet (v3.0.0) using DIP, BIOGRID, HPRD, INTACT, MINT, and BIND protein-protein interaction database sources. Nodes not connected to the largest network are excluded from this view, but the extended view with all nodes is available in S1 Fig. ClusterOne analysis of this network identifies two significantly interconnected clusters (red circles) representing protein complexes corresponding to known biological roles of FANCA in replication and DNA damage response.

To develop a comprehensive protein-protein interaction network (PPIN) for FANCA in pancreas β cells, the experimental conditions for the immunoprecipitation and enrichment of endogenous FANCA and its associated protein complexes were first optimized using 293FT cell lysates. 293FT cells were utilized because they express sufficient amounts of endogenous FANCA protein and obtaining sufficient protein quantities to perform co-immunoprecipitation (co-IP) experiments is readily achievable compared to human islet purifications from donor pancreas or β cell lines. Briefly, cell lysates were generated from 293FT cells in culture and biological duplicate co-IPs were performed using either a non-specific normal rabbit IgG as a negative control or with anti-FANCA antibody. Immunocomplexes were washed, eluted, separated by SDS-PAGE and subjected to in-gel digestion with trypsin. Peptides were eluted and analyzed by liquid chromatography-tandem mass spectrometry (LC-MS/MS) to identify proteins in the sample. High confidence FANCA interactors were distinguished from low confidence and contaminating proteins using non-specific normal rabbit IgG co-IP experiments (n = 2) along with data deposited in the CRAPome (n = 282) [34] as control dataset inputs into the Significance Analysis of Interactome (SAINT) algorithm [35] (**Fig 2A**). A total of 2039 proteins were discovered in the initial FANCA co-IP experiments in 293FT cells by LC-MS/MS with ≥ 2 peptides identified in at least one of the experiments, but only 137 high confidence proteins were identified with a SAINT-determined Bayesian false discovery rate (BFDR) ≤ 0.05 and SAINT score ≥ 0.8 (**Fig 2B** and **S1 Table**). In the two FANCA-specific co-IP experiments in 293FT cells, FANCA was detected by 106 and 127 total spectra in each biological duplicate, indicating successful purification of this protein (**S1 Table**).

Analysis of the functional enrichments present in the 137 high confidence proteins interacting with FANCA was performed using ClueGO interrogation of Reactome Pathways. As expected, both “DNA Repair” (R-HSA:73894, p-value = 2.0E-7) and the “Fanconi Anemia Pathway” (R-HSA:6783310, p-value = 1.1E-5) were enriched in this data set (**Fig 2C**). The enrichment of these two Reactome pathways was driven, in part, by the known interactions with other FA protein family members, including FANCB, FANCG, FANCL, FAAP100, and FAAP20 (**Fig 2B** and **2D**). Known interactions between the 137 FANCA interacting proteins were mapped using BisoGenet [36] (**S1 Fig**). ClusterOne [37] was used to identify significantly interconnected protein complexes, which reveals two clusters of proteins associated with DNA damage and replication proteins (Cluster #1) or the Fanconi Anemia core complex (Cluster #2) (**Fig 2D**). These results confirm known physical associations in the FANCA PPIN dataset and identify interactions that can explain the co-complexes observed.

Previously unrecognized FANCA interactions with proteins associated with the Reactome “DNA Repair” term were also identified, such as HERC2, RFC2/4/5, MSH3, and RAD17, suggesting the potential for additional roles of FANCA in DNA damage response (DDR) beyond those previously reported. The enrichment of other Reactome Pathway terms for the FANCA interacting proteins, such as “Cellular Response to Heat Stress” (R-HSA:3371556, p-value = 1.1E-3) and “Loss of Nlp from Mitotic Centrosomes” (R-HSA:380259, p-value = 1.5E-4), also suggests an expanded role for FANCA beyond its canonical role in the DDR (**Fig 2C**). These results confirm that this purification system can enrich FANCA and known protein interactions, suggesting this method is suitable for implementation in other cell lines.

### FANCA displays differential protein interactions in EndoC-βH3 cells in response to glucose stimulation

**Fig 3:**
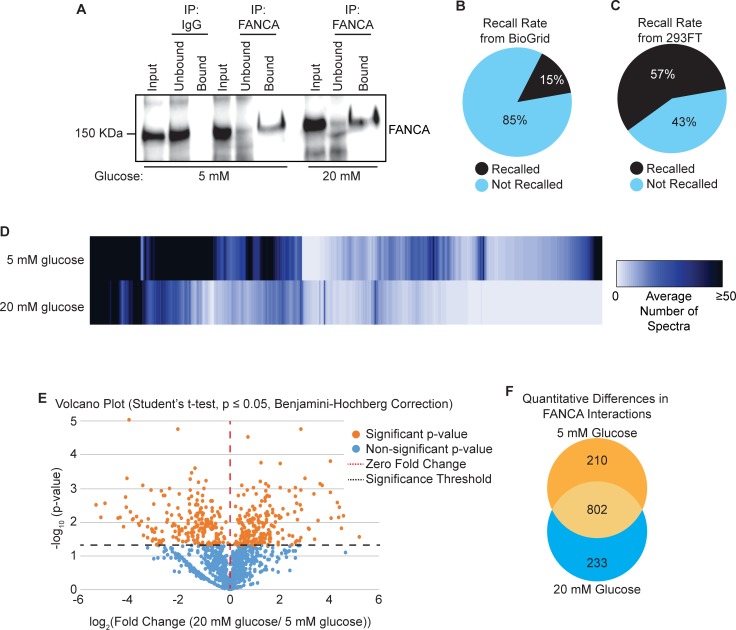
Glucose regulates the FANCA PPIN in EndoC-βH3 cells. **A.** Validation of the specificity and efficiency of the FANCA immunoprecipitation conditions from EndoC-βH3 cells. Co-IP analysis of FANCA in the input, unbound, and bound fraction, after incubation with either 5 mM or 20 mM glucose. **B.** The recall rates of known FANCA protein interactions from the BioGrid database also identified in EndoC-βH3 experiments. **C.** The recall rates of FANCA protein interactions from EndoC-βH3 cells in the 293FT dataset from Fig 2 **D.** Heat map representing the protein expression profiles of FANCA protein interactions in both the 5 mM and 20 mM glucose treatment conditions. Coloring is dependent on the average number of spectra for each protein based on SAINT score. Visualization generated with ProHits-Viz using the results from the SAINT analysis. First pass = 1.0 SAINT score, second pass = 0.9 SAINT score, normalized to FANCA bait. **E.** Volcano plot of FANCA interactors with a SAINT score = 1.0 to identify proteins significantly affected by glucose concentration. Orange nodes represent proteins interacting with FANCA that are significantly affected by glucose concentration (Student’s t-test, Benjamini-Hochberg corrected p-value ≤ 0.05). Blue nodes are proteins whose interactions with FANCA are not significantly affected by glucose levels. **F.** Venn diagram illustrating the number of significantly regulated proteins interacting with FANCA in 5 mM and 20 mM glucose.

Using the conditions optimized in 293FT cells, endogenous FANCA was immunoprecipitated from EndoC-βH3 cells that were cultured in either 5 mM or 20 mM glucose (**Fig 3A**). This experiment identified a total of 3003 proteins in the FANCA PPIN from cells cultured in 5 mM glucose and 2379 proteins from cells cultured in 20 mM glucose with ≥ 2 peptides identified in at least one of the biological duplicate experiments. SAINT was used to assign and rank probabilities of these interactions with FANCA. A total of 1417 proteins from both the 5 mM and 20 mM glucose conditions had a BFDR ≤ 0.05 and SAINT score = 1.0 (**S2 Table**). Of these SAINT-filtered interactions, 15% are recalled from the FANCA PPIN cataloged in the BioGRID database [38] (**Fig 3B**), which is comparable to the recall rates we have previously established for large-scale protein interaction analyses of DNA damage proteins [39]. Because of the availability of the FANCA interactions we identified in 293FT cells, we also analyzed the recall rates from that dataset (**Fig 3C**). The FANCA interactions identified in EndoC-βH3 cells overlapped with 78 of the 137 interactions (57%) identified in the 293FT cells described in **Fig 2**. Many of the FANCA interactions in EndoC-βH3 cells display differential representation by their normalized average spectra between the culture conditions (**Fig 3D**). Statistical analysis using the Student’s t-test corrected with the Benjamini-Hochberg method identified 210 proteins with elevated representation interacting with FANCA in the 5 mM glucose condition, whereas 233 were elevated under the 20 mM glucose condition (**Fig 3E** and **3F**). This result illustrates that the FANCA interactome in EndoC-βH3 cells is both complex and dynamically regulated in response to glucose stimulation.

### Differential FANCA PPIN identifies functional associations dependent upon glucose stimulation

**Fig 4:**
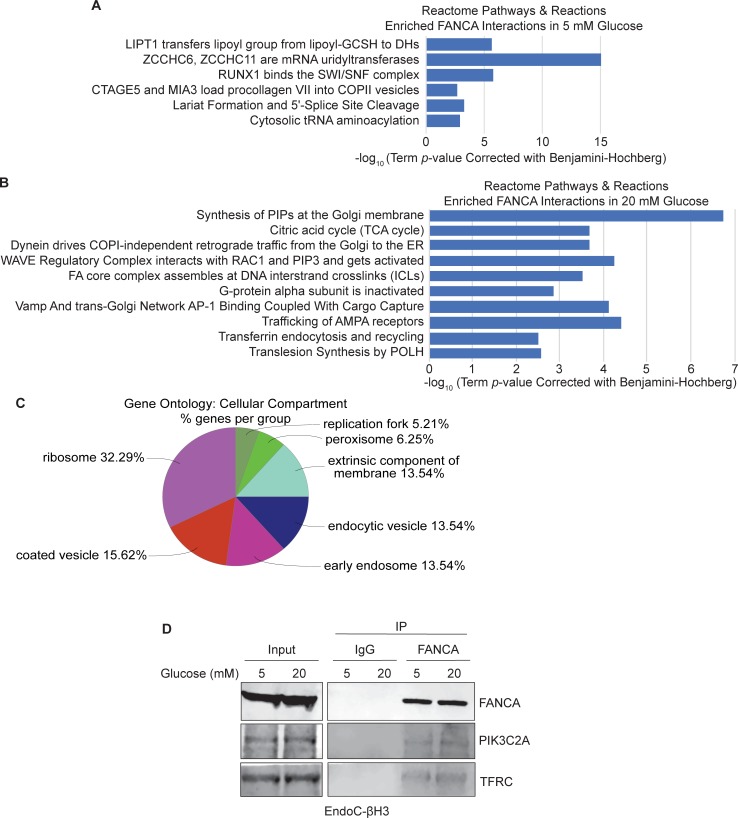
Pathway analysis of FANCA PPIN reveals glucose-dependent associations. ClueGO analysis using Reactome Pathways and Reactions terms for the proteins interacting with FANCA found to be significantly elevated in either 5 mM (n = 210) (**A**) or 20 mM (n = 233) (**B**) glucose conditions. Each term listed in the graph represents the highest scoring term for its group. Complete ClueGO results available in S3 and S4 Tables. **C.** ClueGO analysis of gene ontology terms for cellular compartment for the 233 proteins with significantly elevated interactions with FANCA in 20 mM glucose conditions. Code = All_without_IEA, GO term fusion, GO Tree Interval = 3–5, GO Term Selection: minimum number of proteins = 3, minimum percentage of genes = 4%. Right-sided hypergeometric test (enrichment) with Benjamini-Hochberg p-value correction. **D.** Co-immunoprecipitation of FANCA from EndoC-βH3 cells confirms the interactions identified with PIK3C2A and TFRC by western blot.

The 210 proteins demonstrating elevated interactions with FANCA in the 5 mM glucose condition were analyzed by ClueGO using the Reactome Pathways and Reactions ontology datasets. A total of 33 terms were significantly enriched that could be broadly assigned to 6 different major ontology categories after multiple testing correction (**S3 Table**). The most significant term for each of these groups and its corresponding p-value is displayed in **Fig 4A**. Many of the proteins associated with these Reactome Pathways and Reactions found interacting with FANCA at 5 mM glucose are involved in the regulation of gene expression. SMARCA2, SMARCA4, SMARCC2 are proteins in the SWI/SNF chromatin remodeling complex that regulate nucleosome positioning involved in the regulation of gene transcription. Both SMARCA2 and SMARCA4 are tumor suppressors and mutations are frequently found in cancers, which may suggest an additional link between FANCA mutations and cancer predisposition. The proteins EPRS, FARSB, GARS, MARS, RARS, and WARS are involved in tRNA aminoacylation and implicate FANCA in the regulation of tRNA pools necessary for protein translation. FANCA interactions with spliceosomal complex proteins also suggest a functional role in posttranscriptional regulation.

The FANCA interactions identified in the EndoC-βH3 cell line also suggest the potential for non-canonical protein functions in diverse cellular processes. For instance, FANCA interacts with proteins involved in COPII vesicles involved in the transport of protein cargo from the rough endoplasmic reticulum (ER) to the Golgi apparatus, such as MIA2, MIA3, PREB, SEC24B, SEC24C and HLA-A (**S3 Table**). A ClueGO-based Reactome Pathways and Reactions analysis was also performed on the 233 FANCA interacting proteins significantly elevated in the 20 mM glucose condition (**S4 Table**). Among these 233 proteins, many of the proteins are those involved in Golgi-related pathways and reactions, such as “synthesis of phosphatidylinositol phosphates (PIPs) at the Golgi membrane”, “dynein-driven retrograde traffic from the Golgi to the ER”, and “Vamp and trans-Golgi Network AP-1 Binding Coupled with Cargo Capture” (**Fig 4B**).

We next examined the gene ontology enrichment for cellular compartment terms for the proteins with elevated FANCA associations in 20 mM glucose, which identifies intracellular vesicles as some of the most prominent localizations associated with its interactors (**Fig 4C**). Nuclear localization at replication forks were also identified, confirming previous knowledge of this protein in DNA replication [40]. In addition, “insulin receptor recycling” was a term significantly associated with a group of 4 V-type proton ATPase subunits involved in the acidification of intracellular compartments. These proteins all displayed an increased FANCA association in 20 mM glucose (corrected p-value = 8.3E-04) falling in the same ClueGO group under “transferrin endocytosis and recycling” (**S4 Table**). Validation of the FANCA interactions with Phosphatidylinositol 4-phosphate 3-kinase C2 domain-containing subunit alpha (PIK3C2A) and Transferrin receptor protein 1 (TFRC) was performed using co-immunoprecipitation and western blot analysis (**Fig 4D**). PIK3C2A is localized to the trans-Golgi network where it promotes the synthesis of phosphatidylinositol phosphates (PIPs) and is involved in insulin secretion and signaling [41, 42]. TFRC (also known as CD71) is required for the cellular uptake of iron and regulates the development of erythrocytes and the nervous system [43, 44]. These results suggest varied roles for FANCA outside of its canonical role in ICL repair.

### Glucose stimulation promotes the assembly of the FA core complex

**Fig 5:**
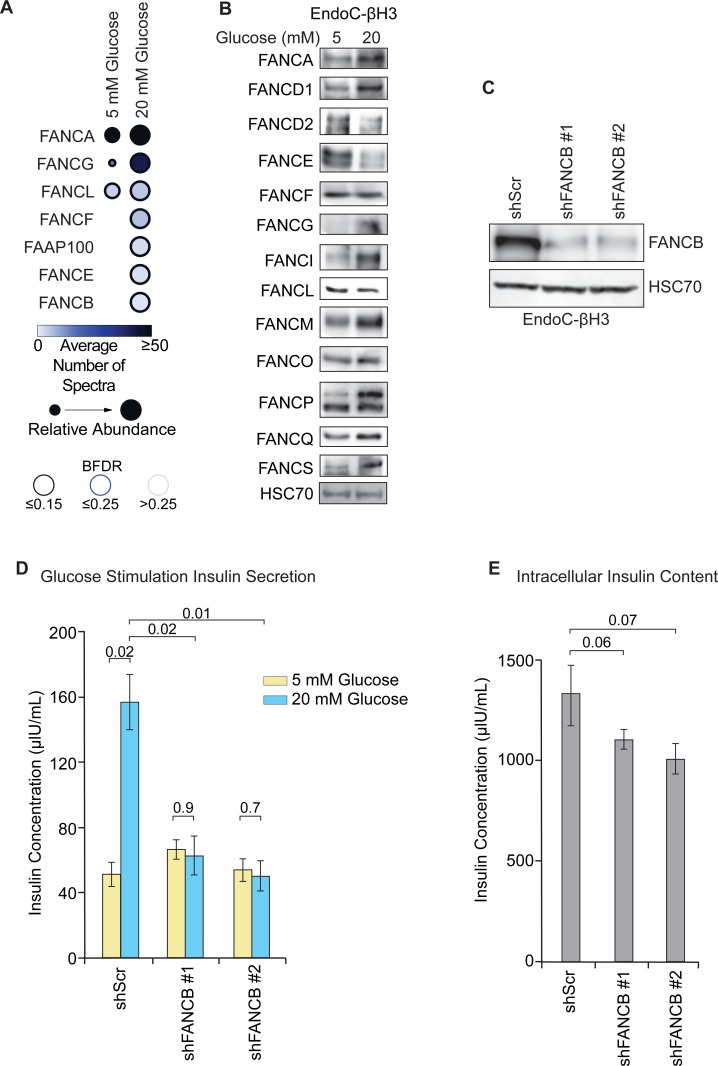
FA core complex assembly and FA protein expression in EndoC-βH3 cells in response to glucose stimulation. **A.** Dot Plot of FA proteins identified in the FANCA co-IP analysis between 5 mM and 20 mM glucose conditions in EndoC-βH3 cells. Dot size represents relative abundance. Dot color indicates the average number of spectra in each experiment. BFDR is represented by the outline shading. **B.** Expression of a panel of FA proteins in EndoC-βH3 cells cultured in either 5 mM or 20 mM glucose for 1 hour determined by western blot. **C.** Confirmation of FANCB knockdown by shRNA (shFANCB) compared to non-targeting scrambled control shRNA (shScr) in EndoC-βH3 cells. **D.** GSIS profiles in EndoC-βH3 shScr or shFANCB transduced cells. Paired two-tailed Student’s t-test *p*-values are displayed in the indicated comparisons above the graph. n = 3, mean +/- SEM, n.s. = not statistically significant. **E.** Intracellular insulin content of shScr or shFANCB EndoC-βH3 cells used in the GSIS assays in panel C. Paired two-tailed Student’s t-test *p*-values are displayed in the indicated comparisons above the graph. n = 3, mean +/- SEM.

One of the Reactome Pathway terms significantly enriched in response to 20 mM glucose stimulation in the FANCA interaction analysis was “FA core complex assembles at DNA interstrand crosslinks (ICLs)” (**Fig 4B** and **S4 Table**). This result suggests that glucose stimulation leads the active assembly of the FA repair complex, which is a process normally induced by DNA damage. Examination of the FA proteins found in the FANCA interaction data from the mass spectrometry experiments reveals that glucose stimulation increases the levels of FANCG, FANCL, FANCF, FAAP100, FANCE and FANCB interacting with FANCA (**Fig 5A**). Notably, FANCB, FANCE, FANCF, and FAAP100 were not identified or did not pass SAINT filtering in the 5 mM glucose condition but were identifiable in complex with FANCA after glucose stimulation at 20 mM (**Fig 5A**). This suggests that the FA core complex is actively assembling in response to glucose stimulation in EndoC-βH3 cells.

We next sought to examine the effects of increased glucose concentration on the expression of a panel of FA proteins in EndoC-βH3 cells to determine if the assembly of the FA complex correlated with protein expression. Glucose stimulation-induced higher expression levels for FANCA, FANCD1, FANCG, FANCI, FANCM, FANCP, FANCQ, and FANCS (**Fig 5B**). However, lowered expression of FANCD2 and FANCE were also observed in 20 mM glucose compared to 5 mM control. No effect was seen on the expression of FANCF, FANCL, or FANCO. These results indicate that expression of most FA proteins is actively regulated in response to glucose stimulation. Because the glucose stimulation is only performed for 1 h, this regulation occurs quite rapidly. The increase in FANCD1 representation in the FANCA pulldown identified by mass spectrometry might be attributable to elevated protein expression following glucose stimulation (**Fig 5B**). However, the other FA proteins in complex with FANCA at 20 mM glucose either do not demonstrate changes in protein expression (FANCE and FANCL) or are repressed (FANCE) (**Fig 5B**), suggesting qualitatively that their association with FANCA may represent the active assembly of the FA complex.

The assembly of the FA complex following glucose stimulation suggests other FA proteins could also participate in GSIS. To evaluate the role other FA complex proteins in GSIS, we performed a shRNA-mediated knockdown of FANCB in EndoC-βH3 cells (**Fig 5C**). Like knockdown of FANCA, the knockdown of FANCB also led to a decrease in GSIS (**Fig 5D**). The impaired GSIS was not associated with a decrease in intracellular insulin pools (**Fig 5E**). It should be noted that pancreas endocrinopathies are not restricted to a single FA complementation group, suggesting mutations in different FA genes can lead to beta cell dysfunction. This is reflected in our results where inhibition of either FANCA or FANCB expression leads to defects in GSIS.

### GSIS requires ROS, which also activates the DDR in EndoC-βH3 cells

**Fig 6:**
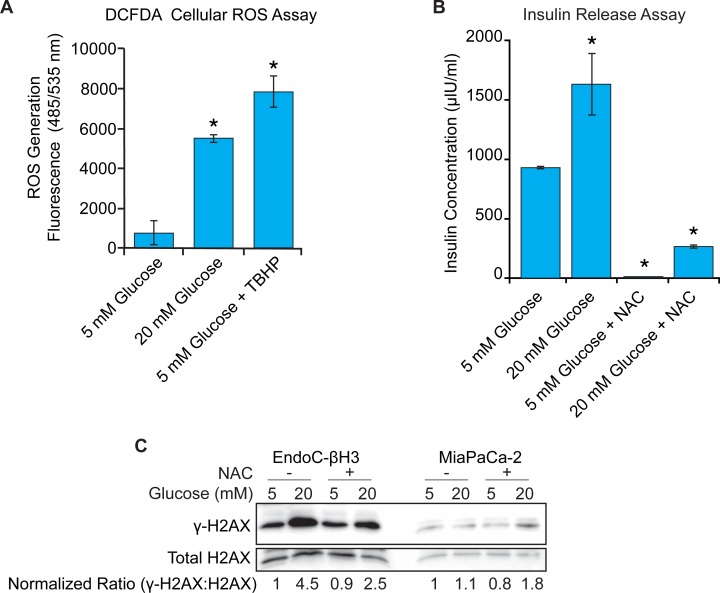
ROS production is increased in response to glucose, necessary for GSIS, and increases DNA damage in EndoC-βH3 cells. **A.** EndoC-βH3 cells were labeled with DCFDA (20 μM) and then cultured an additional 3 hours at 5 mM glucose with or without 50 μM tert-butyl hydrogen peroxide (TBHP) or at 20 mM glucose. n = 3, mean +/- SEM, p ≤ 0.05, paired two-tailed Student’s t-test. TBHP mimics ROS activity to oxidize DCFDA to fluorescent DCF and is used as a positive control. **B.** GSIS profiles of EndoC-βH3 cells with and without NAC treatment. Asterisk (*) denotes statistically significant difference compared to 5 mM glucose without NAC. n = 3, mean +/- SEM, p ≤ 0.05, paired two-tailed Student’s t-test. **C.** Evaluation of cellular DNA damage levels by western blot for γ-H2AX and total H2AX in response to 5 mM and 20 mM glucose with and without NAC. The normalized ratio of γ-H2AX: H2AX is displayed below the blots determined by Li-COR Image Studio.

In rodent systems, it has been demonstrated that ROS is produced in β cells and is necessary to promote insulin secretion [45, 46]. We confirmed that ROS levels are increased in response to glucose stimulation in the human EndoC-βH3 cells (**Fig 6A**). Pre-incubation with the ROS scavenger N-acetylcysteine (NAC) for 1 h abolished most insulin secretion at both 5 mM and 20 mM glucose conditions (**Fig 6B**). These results reveal that ROS is elevated in response to glucose and is required for the secretion of insulin from the EndoC-βH3 cells.

ROS damage both the DNA nucleobases and the sugar phosphate backbone leading to many different lesions, including interstrand crosslinks that require the FA pathway for repair [47]. Therefore, it is possible that glucose stimulation could be inducing DNA damage through ROS production. Thus, we examined the status of γ-H2AX expression, a marker for DNA damage, and found that elevated glucose levels promote DNA damage in EndoC-βH3 cells (**Fig 6C**). When cells were stimulated with 20 mM glucose and treated with NAC, γ-H2AX levels were reduced in comparison to cells not receiving NAC. These results contrast with glucose-insensitive MiaPaCa-2 pancreatic ductal adenocarcinoma cells that do not exhibit glucose stimulated DNA damage in the presence or absence of NAC. Together, these results suggest an association between glucose-induced ROS production necessary for insulin release and increased DNA damage specifically in pancreas β cells.

## Discussion

The development of diabetes in FA patients is likely multifactorial. Two potential functions of FA proteins in β cells that when disrupted in FA patients leads to hyperglycemia are: 1) FA proteins are necessary for GSIS; and 2) FA proteins reduce β cell susceptibility to glucose toxicity. The results from this study suggest a model where FA proteins may function in both processes. Knockdown of FANCA or FANCB prevents GSIS in an assay that is performed in an hour, suggesting loss of FANCA or FANCB has immediate impacts on GSIS not attributable to glucose toxicity. Glucose toxicity involves cell death mechanisms that would act on a much longer time scale than the GSIS assay. However, our findings also suggest that glucose stimulation induces the activation of the DDR in an ROS-dependent manner. This data suggests that β cell dependence on ROS, as a factor necessary for GSIS, exposes islet cells to elevated genotoxic stress. In the absence of an intact FA repair pathway, ROS could lead to the accumulation of DNA damage and cell death. Thus, it is possible that the initial hyperglycemia experienced in FA patients may be attributable to a defect in GSIS that is further compounded by increased glucose toxicity of β cells triggered by ROS-induced DNA damage that cannot be repaired. Our data demonstrates that loss of either FANCA or FANCB in beta cells impairs GSIS, and glucose stimulation promotes the assembly of the FA complex, suggesting this complex may have unrecognized roles in beta cell-specific functions.

It should be noted that this study does not attribute the assembly of the FA complex to the induction of the DNA damage observed upon glucose stimulation. In the western blot analysis, we did not observe mono-ubiquitination of either FANCI or FANCD2, which are markers of an active ICL repair response through the FA pathway, following glucose stimulation. Furthermore, the EndoC-βH3 cells are in a growth arrested terminal differentiation state following addition of 4-OHT and excision of the immortalizing transgenes, which would attenuate DNA repair processes [48, 49]. Thus, FA complex assembly and the induction of DNA damage could be either dependent or independent events caused by elevated glucose concentrations.

Decreased response to oxidative stress in FA patients is well-documented and antioxidant therapy has been postulated as a potential treatment for these patients, but a definitive clinical benefit has not been demonstrated [50]. The association between FA dysfunction, ROS and GSIS in beta cells is also unclear. It was previously shown that beta islet cells obtained from fanca-/- mice did not have a significant increase in cellular ROS compared to wild type controls. Cellular ROS levels in insulin responsive liver, muscle, and fat tissues were also unaffected by *fanca* status regardless of being fed either a normal or high-fat diet [18]. However, elevated ROS levels were observed after TNF-alpha treatment in *fanca-/-* beta islet cells [18]. Elevated levels of TNF-alpha observed in FA patients could increase oxidative stress through inflammation and ROS production [51]. Treatment of *fanca*-/- mice with the ROS scavenger Quercetin can mitigate diabetes and obesity prone phenotypes in part through regulation of insulin receptor signaling [18]. Moving forward, it will be important to delineate the process involving ROS production and the assembly of the FA complex induced by glucose stimulation.

The work presented in this study is the first, to our knowledge, to investigate the endogenous protein interactions with FANCA in endocrine pancreas β cells and how they are altered upon glucose stimulation. The FANCA PPIN analysis revealed many novel associations impacted by glucose stimulation including Golgi functions. Interestingly, the Golgi packaging and transport of insulin prior to exocytosis is a well-established process [52, 53]. Recent evidence also suggests that FA proteins, including FANCA, regulate mitophagy in a process distinct from their functions in DNA damage repair [54]. Autophagy itself plays an important role in the regulation of β cell function, and the inhibition of this process results in the development of type 2 diabetes [55]. Both autophagy and mitophagy are multistep processes utilizing autophagosomal membranes that can carry membrane contents from intracellular organelles including the Golgi apparatus [56]. It should be noted that autophagy proteins were found to interact with FANCA in our screen of β cells, including ATG2A, ATG4B, and ATG9A. Further evaluation of the role of FA proteins in cellular processes involving intracellular vesicles, such as autophagy and insulin secretory pathways, could shed light on non-canonical functions of these proteins that contribute to the endocrinopathies in FA patients.

The FANCA PPIN developed here could shed light on some of these processes. For instance, a correlation has been observed between serum ferritin levels and insulin resistance in FA patients [57]. Our data suggests that FANCA interacts with proteins involved in transferrin endocytosis and recycling, such as TFRC, following glucose stimulation. Defects in FANCA-mediated regulation of transferrin endocytosis and recycling in FA patients could lead to the accumulation of serum ferritin and contribute to the elevated oxidative stress in these patients. Furthermore, defects in this pathway caused by loss of FANCA could explain why FA patients suffer from iron overload and suggests that transfusions used to treat the anemia might not be the only cause of this toxicity. Working with a complete PPIN for FANCA will allow additional research into how the DNA damage-dependent and independent functions of this protein contributes to the manifestation of the disease that could inform better treatment strategies for both cancers and co-morbidities that are found in FA patients.

In conclusion, this study identifies FANCA and FANCB as modifiers of GSIS, glucose stimulates the assembly of the FA core complex and establishes a β cell specific FANCA PPIN regulated by glucose stimulation. Together, these results provide new insights into the role of FANCA in β cells that increase our understanding of FA-associated β cell dysfunction. Working with a complete PPIN for FANCA will allow additional research into how the DNA damage-dependent and independent functions of this protein contributes to the manifestation of the disease that could inform better treatment strategies for both cancers and co-morbidities that are found in FA patients.

## Supporting information

S1 Fig Extended figure related to Fig 2D. (PDF)

S1 Table FANCA SAINT output from 293FT lysate. Column descriptions: Bait: bait identifier; Prey: prey identifier; PreyGene: additional prey identifier; Spec: spectral counts for the bait-prey pair; SpecSum: sum of the spectral counts; AvgSpec: average spectral counts over replicates; NumReplicate: number of replicate purifications for the given bait; ctrlCounts: spectral counts in the negative controls; AvgP: main probability score; MaxP: maximal probability score of the interaction over replicates; TopoAvgP: topology-aware probability score incorporating known interaction data; TopoMaxP: topology-aware maximal probability score over replicates; SaintScore: larger of AvgP and TopoAvgP; logOddsScore: log likelihood ratio of the observed data over the expected value; FoldChange: average spectral count in test interaction divided by the average in controls; FDR: Bayesian false discovery rate. High-confidence interactions considered those with a SAINT-determined BFDR ≤ 0.05 and SAINT score ≥ 0.8.
(XLSX)

S2 Table FANCA SAINT output from EndoC-βH3 cells. The same column descriptions as those provided in S1 Table. High-confidence interactions considered those with a SAINT-determined BFDR ≤ 0.05 and SAINT score = 1.0.
(XLSX)

S3 Table ClueGO result table for 5mM glucose using reactome reactions and pathways. Output file from ClueGO for the 210 proteins with elevated representation in 5 mM glucose conditions.
(XLSX)

S4 Table ClueGO result table for 20 mM glucose using reactome reactions and pathways. Output file from ClueGO for the 233 proteins with elevated representation in 20 mM glucose conditions.
(XLSX)
