# Supplementary figures and images for: Delineating the role of FANCA in glucose-stimulated insulin secretion in β cells through its protein interactome
Source: PLoS One. 2019 Aug 28;14(8):e0220568. doi: 10.1371/journal.pone.0220568 (PMC6713327; doi:10.1371/journal.pone.0220568)

- FANCA (Bait)
- FANCA Interaction (Database)
- Novel FANCA Interaction

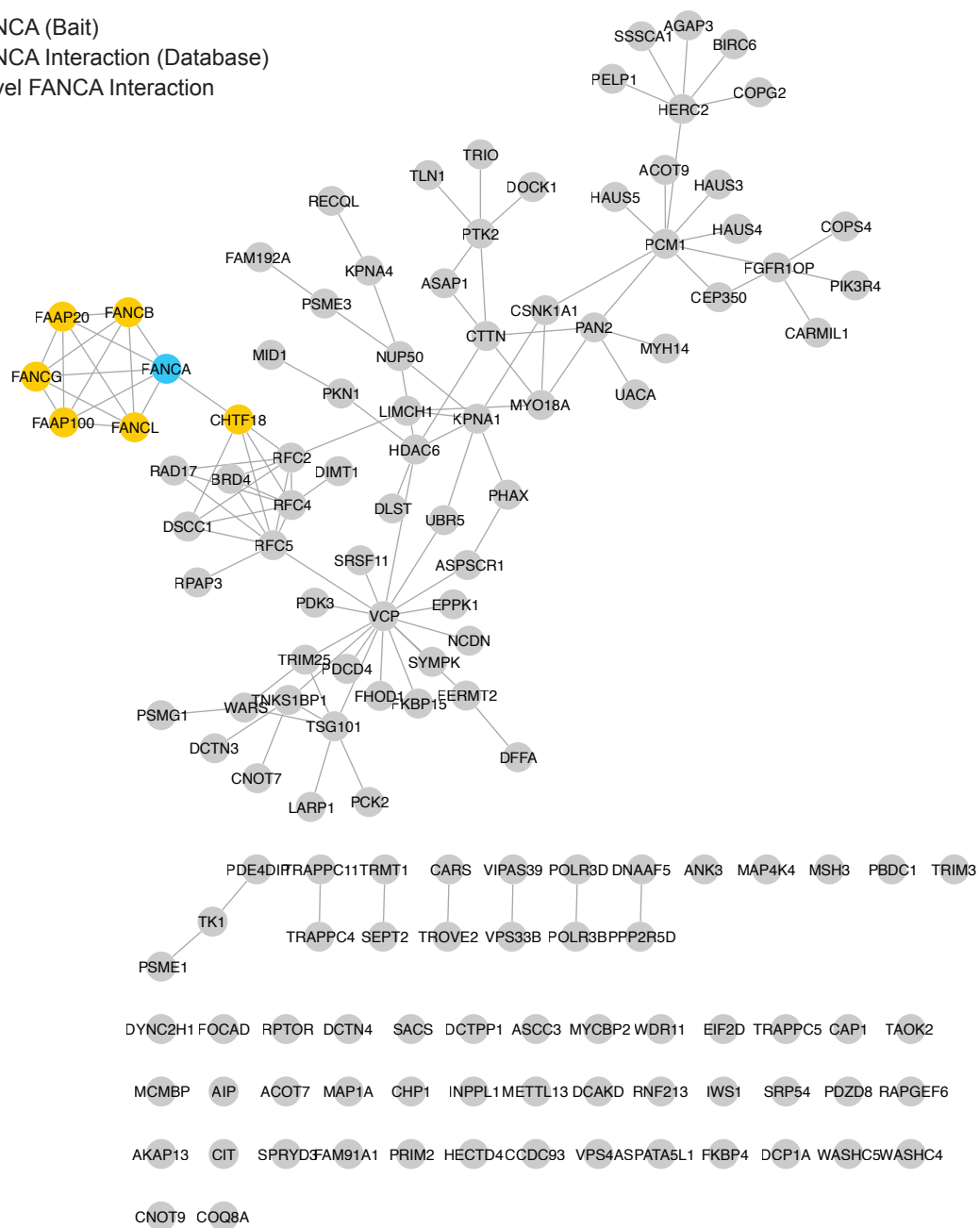

Supplement: S1 Fig — (PDF) [file pone.0220568.s001.pdf]
